# Physicochemical Perspective of Biological Heterogeneity

**DOI:** 10.1021/acsphyschemau.3c00079

**Published:** 2024-04-06

**Authors:** Karina Kwapiszewska

**Affiliations:** Institute of Physical Chemistry, Polish Academy of Sciences, Kasprzaka 44/52, Warsaw 01-224, Poland

**Keywords:** biochemical reactions in situ, physical properties of cells, intracellular environment, biological heterogeneity, biochemical networks

## Abstract

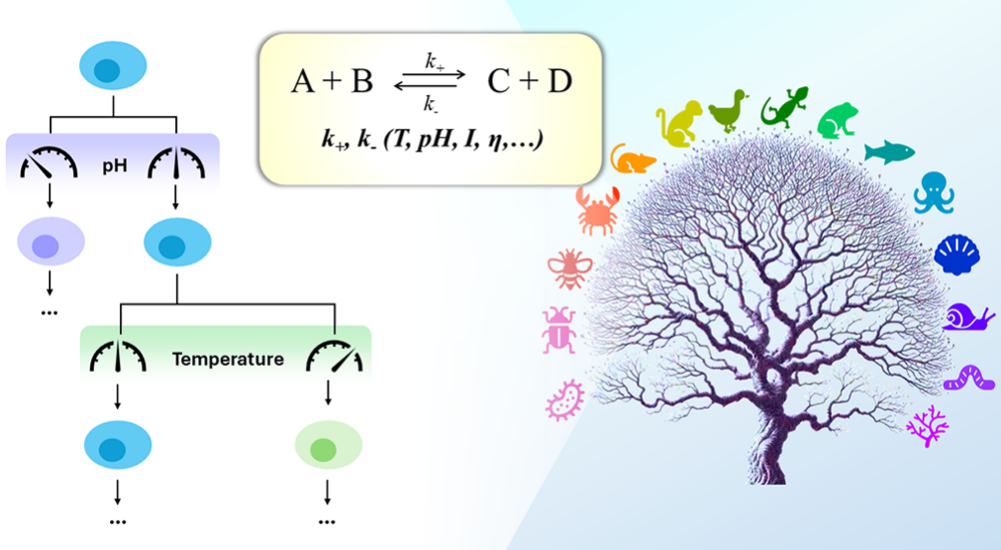

The vast majority of chemical processes that govern our lives occur within living cells. At the core of every life process, such as gene expression or metabolism, are chemical reactions that follow the fundamental laws of chemical kinetics and thermodynamics. Understanding these reactions and the factors that govern them is particularly important for the life sciences. The physicochemical environment inside cells, which can vary between cells and organisms, significantly impacts various biochemical reactions and increases the extent of population heterogeneity. This paper discusses using physical chemistry approaches for biological studies, including methods for studying reactions inside cells and monitoring their conditions. The potential for development in this field and possible new research areas are highlighted. By applying physical chemistry methodology to biochemistry *in vivo*, we may gain new insights into biology, potentially leading to new ways of controlling biochemical reactions.

## Introduction

Heterogeneity is a common characteristic of any biological sample. Even apparently similar cell types can display distinct genetic and phenotypic differences within populations. As complexity increases, such as in tissues, organisms, or populations of whole organisms, so does heterogeneity. This has significant implications for the predictability of biological studies. Good research practice involves proving any hypothesis in multiple experimental systems. When dealing with heterogeneous biological systems, the number of experiments should be increased, and many controls should be added to distinguish between the studied effect and the quality of the biological sample. Therefore, biological experiments are costly, time-consuming, and extremely difficult to interpret.

**Figure 1:**
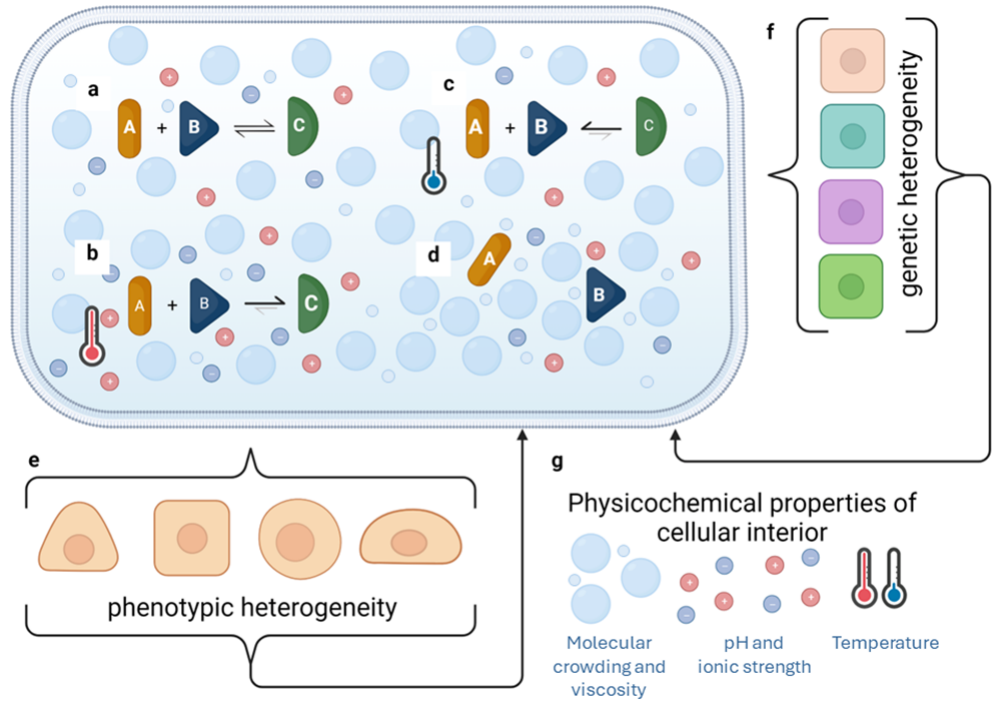
There is a complex relationship between the cytoplasm’s and nucleoplasm’s physicochemical properties and the different levels of biological heterogeneity. (a) Life processes can be represented as equilibria, with reactions depending on the environment surrounding them. (b, c) Changes in temperature, ions, or macromolecules can alter this balance, favoring substrates or products. (d) In some cases, increased viscosity or steric restrictions can even prevent reactions from occurring. All of these effects (a-d) can occur in different compartments of the same cell. (e, f) Depending on the type of reaction affected, such as DNA processing or gene expression/metabolic pathways, slight changes in pH or temperature can result in phenotypic or genetic heterogeneity in a population of cells. (g) This, in turn, leads to further alterations in molecular composition and regulation of pH, ion concentrations, or temperature, which can further influence diversity in the outcomes of chemical reactions.

On the other hand, physical chemists focus on studying homogeneous samples of molecules under well-defined and controlled conditions. This approach enables them to understand the chemical phenomena at their very core with relatively fewer experiments. However, most chemical processes that guide our lives occur within the interior of living cells. Therefore, intracellular conditions, which vary from cell to cell and organism to organism, significantly impact further biochemical reactions, increasing the extent of population heterogeneity (Figure 1). Hence, studying biochemical reactions requires simultaneous monitoring of intracellular conditions. Local physicochemical properties of cytosol or nucleosol can influence DNA processing, mRNA processing, translation, protein function, or transport—thus impacting every aspect of cell function. Correlating the course of biochemical reactions with local conditions in individual cells can be beneficial for understanding biological phenomena, such as gene expression, protein activity, or metabolism.

This work discusses the use of physical chemistry approaches for biological studies. The article reviews methods for studying reactions inside cells and ways to monitor their conditions. Although there is currently a technological gap in controlling the environment inside cells, the article highlights the potential for development in this field. Additionally, the paper outlines possible new areas of research. We may gain new biological insights by applying physical chemistry methodology to biochemistry *in vivo*. These efforts could improve our understanding and lead to new ways of controlling biochemical reactions, with the potential for use in biotechnology or personalized medicine.

## Physical Chemistry—Finding Models Describing Nature

At its core, physical chemistry seeks to understand and explain matter’s physical and chemical properties and the changes it undergoes. Theoretical models in physical chemistry often involve the application of mathematical formulations, quantum mechanics, thermodynamics, and statistical mechanics to interpret experimental observations and predict the behavior of matter under different conditions. For example, in chemical kinetics, theoretical models can predict reaction rates based on the energy profiles of reactants and transition states, allowing researchers to comprehend the temporal evolution of chemical reactions.^1^ Furthermore, physical chemistry plays a pivotal role in developing models that describe the behavior of materials at the molecular level, aiding advancements in fields such as materials science and nanotechnology.^2,3^ Theoretical models in physical chemistry have been instrumental in elucidating molecular structures, predicting spectroscopic properties, and understanding the thermodynamic stability of materials.^4^ From electronic structure calculations to molecular dynamics simulations, physical chemistry provides a robust framework for constructing models that guide researchers in comprehending and manipulating the intricate molecular processes underlying a wide array of natural phenomena. These models not only deepen our understanding of fundamental scientific principles but also contribute to technological innovations and practical applications across diverse scientific disciplines.^4^

However, this wide range of possibilities narrows with the increasing complexity of the studied system. Thus, the majority of theories in physical chemistry start with simplified system models. An extreme example can be the ideal gas model—a theoretical concept that assumes a gas is composed of particles with negligible volume, experiencing no intermolecular forces except during collisions, and whose behavior is described by the ideal gas law, *PV* = *nRT*.^5^ It serves as a simplified model for understanding the fundamental principles governing the behavior of gases under specific conditions. Still, it does not describe any of the systems existing in nature. Natural systems are full of interactions between the molecules. For example, when it comes to modeling chemical reactions, not only reagents interact with each other. The effect of the solvent—inherent surroundings of the molecules—is an essential part of theories on chemical reaction rates.^6,7^ More recent research shows that buffer ingredients, or even container material, can influence the model as well.^8−10^ Neglecting particular terms of the models should be preceded with detailed investigations based on experiments in well-controlled conditions.^11^ This extensive control over the experimental environment is accessible in chemical laboratories. Regarding biological systems, only a few parameters can be controlled.^12^

## Heterogeneity in Biology

Biological systems are highly heterogeneous. The diversity and variability observed within biological systems at various levels range from molecular and cellular to organismal and ecological scales.^13^ This diversity manifests in differences among individual cells, organisms, or populations, contributing to the complexity of biological systems. At the molecular and cellular levels, genetic variations, epigenetic modifications, and stochastic processes lead to diverse phenotypes even among genetically identical cells.^14^ This intracellular heterogeneity plays a crucial role in cellular functions, responses to stimuli, and the emergence of distinct cell types within tissues.^15^

On a broader scale, intercellular and organismal heterogeneity are evident in the diverse cell types, tissues, and organs that collectively form an organism. Going further, organisms of the same species vary from molecular to phenotypical level.^16^ Understanding and characterizing heterogeneity in biology has become increasingly important in fields like personalized medicine, where individual variations in genetic makeup and cellular behavior can impact disease susceptibility, progression, and treatment responses.^17^

At the molecular level, random events, such as mutations during DNA replication or genetic recombination, introduce variability in the genetic code. These stochastic processes contribute to the generation of genetic diversity, which serves as the raw material for evolution.^18^ Stochastic events can influence cellular processes, gene expression, and protein folding at the phenotypic level, leading to diverse phenotypic outcomes even among genetically identical individuals.^17^ Looking closer, apparently insignificant intracellular fluctuations, such as thermal or pH changes, can impact biological outcomes.^19^ Moreover, environmental fluctuations, chance events during development, and the inherent probabilistic nature of biochemical reactions all contribute to the emergence of diverse biological traits. Understanding the impact of stochasticity on molecular and phenotypic diversity is crucial for unraveling the complexities of evolutionary processes and the adaptability of living organisms in response to changing environments.

## Key Factors in Biochemistry

When investigating the factors that impact biochemical reactions, we typically focus on those that differentiate biochemistry from classical chemistry. These factors include the primary role of enzymatic catalysis and the reliance on weak interactions. Enzymes play a crucial role in biochemical reactions as they efficiently catalyze reactions and are more cost-effective and specific than chemical catalysts.^20^ On the other hand, the presence of weak intermolecular interactions, such as noncovalent or van der Waals forces, contributes to the extreme efficiency and specificity observed in biological systems.^21^ Still, enzyme-driven reactions in biochemistry follow the laws of kinetics and thermodynamics, which means that factors such as pH, ionic strength, temperature, and the presence of other molecules (macromolecular crowding and viscosity) strongly influence the reaction rates and yields. Therefore, investigating physicochemical factors can provide valuable insights into intracellular biochemistry.

### Temperature

Since the Arrhenius equation was derived, it is widely known that temperature significantly impacts the rate of chemical reactions. Within living cells another dimension is added to this dependence, as most reactions are catalyzed by temperature-sensitive enzymes.^22,23^ Human cells need a narrow temperature range for optimal growth and function (∼37 °C). The activity of enzymes is effectively inhibited at low temperatures, leading to thorough metabolism quenching. On the other hand, elevated temperatures–usually beneficial for chemical reactions–can lead to cellular stress and disrupt normal physiological functions in human cells. Excessive heat can induce protein denaturation, compromising the structure and function of essential cellular proteins. Prolonged exposure to elevated temperatures may trigger cellular damage, impair enzymatic activities, and ultimately compromise the overall health and viability of human cells.^24,25^ Thus, our bodies maintain thermal homeostasis–stable temperature at an optimal level for biochemical reactions.^23^

However, “stable” at the macroscale can mean heterogeneous at the micro- and nanoscale. Also, lower local energy fluctuation can affect cellular function, as energy is exchanged with biomolecules in many intracellular reactions. It was found that temperature fluctuations can be observed even within single cells.^26^ This process has led to the concept of “thermal signaling,” which is the process by which heat is released through intracellular biochemical reactions and influences cellular functions across different spatial scales, from molecules to individual organisms.^26^ It involves releasing heat through various biological processes and biomolecules, such as ATP/GTP hydrolysis and biopolymer complex formation and disassembly.

### pH

Intracellular pH plays a crucial role in various cellular processes, influencing cell growth, metabolism, cytoskeleton polymerization, and membrane transport. Living organisms employ different mechanisms to control pH and ensure proper cellular functions. One key player is the bicarbonate ion (HCO_3_^–^) system, where cells can release or take up bicarbonate to maintain a pH balance.^27^ Additionally, cells utilize various membrane transport proteins, such as proton pumps and ion exchangers, to regulate the concentrations of hydrogen ions (H+) and other ions across the cell membrane.^28^ Intracellular buffering systems involving molecules like proteins and phosphates also play a crucial role in preventing rapid changes in pH by absorbing or releasing protons as needed.^29^ These mechanisms enable cells to control their internal pH tightly and create an optimal environment for cellular processes. This is important for cellular homeostasis, as altered pH can dramatically affect protein conformation and enzyme activity.^30^ Moreover, pH fluctuations were found to impact alternative splicing, mitochondria, plasma membrane, phase separation, and the associated molecular pathways essential for somatic cell reprogramming and differentiation of pluripotent stem cells.^31^

### Ionic Strength and Osmotic Pressure

Ionic strength (IS) influences macromolecular interactions, cell proliferation, survival, size control, protein aggregation, and enzyme activity.^32^ It is also strictly connected with osmotic homeostasis, which refers to maintaining a stable internal environment in response to changes in osmotic pressure. At the cellular level, regulating osmotic homeostasis involves maintaining intracellular water balance and regulating ion concentrations, such as potassium (K^+^) and magnesium (Mg^2+^).^33^ It was found that changes in ionic strength can stabilize or destabilize protein–protein interactions, affecting molecular pathways.^34^ In the context of Alzheimer’s disease, changes in the ionic environment can significantly affect the interactions between amyloid beta (Aβ) and mitochondrial proteins, potentially contributing to mitochondrial dysfunction.^35^

### Viscosity

At the single-cell level, “live processes” encompass a set of interactions and reactions among biomolecules, often driven by molecular mobility. While only a small fraction of intracellular transport is force-driven (active transport, i.e., ATP dependent), free diffusion (Brownian motion) predominantly influences intracellular biomolecular displacements.^36^ Biochemical reactions are diffusion-limited, with rates dependent on diffusion coefficients, which, according to the Stokes-Sutherland-Einstein relation, inversely rely on hydrodynamic drag *f* = 6πη*r*, where *r* is the hydrodynamic radius of a molecule and η is viscosity.^37^ In the case of the cellular interior, effective viscosity should be considered, as it results from solvent viscosity and various macromolecules influencing the motion and interactions of molecules (macromolecular crowding).^38^ This effective intracellular viscosity is a crucial factor affecting biochemical reactions in living cells. Research studies have found a connection between cellular state and effective viscosity. It has been observed that when intracellular crowding is artificially increased, eukaryotic cells undergo damage, programmed cell death, and stress response.^39^

On the other hand, it was observed that apoptosis is accompanied by an increase in internal viscosity.^40^ HeLa cell viscosity, on the other hand, remains stable despite significant structural changes during the cell cycle.^41^ Furthermore, it has been shown that the nanoscale intracellular viscosity is length-scale dependent due to crowding, and its profile is conserved among diverse human cell lines,^42^ which suggests its physiological importance (see next chapter for more details). The nanoviscosity values for length scales less than 1 nm are unexpectedly low, comparable to solvent viscosity.

## Detection Possibilities

To understand heterogeneity in intracellular dynamics comprehensively, it is crucial to have a two-way approach to monitoring. First, we should map spatiotemporal quantities such as temperature, ion concentrations, or viscosity to gain insights into the environment in which biochemical reactions occur. Second, we should observe the reactions themselves as, based on current knowledge, we cannot predict how a given process would unfold in the complex intracellular space.

### Monitoring Intracellular Environment

Numerous techniques have been used to study intracellular dynamics and fluctuations. Most of these methods use optical detection, which is considered less invasive for the cells being studied. Moreover, fluorescent-based microscopy provides multidimensional cell mapping with resolution not achievable by other methods.^43^ Fluorescent thermosensors are commonly used to monitor intracellular temperature. Different designs of such thermosensors have been developed so far. One strategy involves a combination of thermoresponsive domains with fluorescent molecules (protein or small molecules), allowing for real-time temperature monitoring and distinguishing organelle-specific thermogenesis.^44^ Thermoresponsive domains can be based on polymers,^45^ fluorescent gold nanoclusters,^46^ or proteins.^44^ Genetically encoded (protein-based) nanosensors enable organelle-specific temperature measurement through fluorescence changes, which was applied to study cell-cycle-dependent temperature responses and applications in hyperthermia.^47^ Alternatively, the fluorescent molecule can be a sensor itself when the photophysical properties of a small molecule depend on the temperature. Such fluorescent thermosensors can be conjugated with organelle-targeting domains for site-specific temperature monitoring.^48,49^ These diverse approaches contribute to understanding temperature dynamics within living cells at the microscale. However, despite great promise, intracellular thermosensors face numerous limitations, which were reviewed elsewhere.^50^

The first—and most widespread—way of measuring ion concentrations in cells is Förster resonance energy transfer (FRET). It involves the energy transfer between two fluorophores, where the emission of one fluorophore (donor) is transferred to another fluorophore (acceptor) in close proximity. This energy transfer can be detected as a change in fluorescence intensity or spectrum. FRET-based ion-specific biosensors detect ions like Ca^2+^, K^+^, Na^+^, Cu^+^, Zn^2+^, and Cl^–^.^51^ Among those, protein-based ion sensors pose positively and negatively charged α-helix and offer sensitivity in the micro- to millimolar concentration range.^52^ FRET-based sensors are popular tools for measuring ion strength, especially in neuroscience for calcium imaging. While FRET methods for estimating ionic strength face challenges like low signal-to-noise ratio and reduced dynamic range, bioluminescence systems, particularly Renilla luciferase or other luciferases, provide a competitive solution with high sensitivity and a broad dynamic range. For instance, the mNeonGreen Nano Luciferase fusion protein enables monitoring ionic strength alterations at the mM scale in cell lysates and live cells.^53^

Another quantity that can be measured using fluorescence-based methods is viscosity. Measuring intracellular viscosity poses a challenge due to its heterogeneity across sites and length scales.^42,54−57^ Nanoviscosity (also called microviscosity) at length scales below 1 nm can be measured using fluorescent molecular rotors, changing their spectral properties depending on rotational speed.^40,58^ Intracellular viscosity at scales over 1 nm is indirectly measured by assessing the diffusion coefficients of intracellular probes through techniques like Fluorescence Recovery After Photobleaching (FRAP) and Fluorescence Correlation Spectroscopy (FCS).^42,55,56^ Systematic studies reveal that objects of different sizes experience different viscosity, influenced by the complex composition of cytoplasm.^42^ The length-scale dependent viscosity model (LSDV) presents a promising tool for interpreting molecular mobility data and has applications in quantifying protein oligomerization, cellular uptake of drugs and macromolecules, and drug-target interactions *in situ*.^59−62^*R*_H_ and ξ parameters characterize the LSDV model, reflecting the length scales of the complex fluid structure. Surprisingly, various human cells share similar values of *R*_H_ and ξ, highlighting the model’s broad applicability.^42^

The pH also varies across organelles and cell types, and measuring it inside the cell involves techniques such as nuclear magnetic resonance, fluorescence spectroscopy, and microelectrodes.^63^ As mentioned above, fluorescent techniques, mainly using pH-sensitive molecules, have become a common and effective method for high-sensitivity, fast-response, and nondestructive pH testing. Over the years, numerous pH indicators, including fluorescein derivatives, benzoxanthene dyes, and cyanine-based indicators, have been developed.^64^ DNA-based nanoswitches designed for biosensing and imaging, activated by changes in pH, offer promising solutions for *in vivo* monitoring.^65^ Additionally, light activation using near-infrared (NIR) light provides a noninvasive and temporally programmable approach for controlling pH-sensitive nanodevices in live cells, showcasing potential clinical applications.^66^

### Monitoring Propagation of Biochemical Reactions *In Situ*

Monitoring biochemical reactions in cells is challenging due to restricted volume, limited accessibility to the reaction site, as well as highly complicated background. There have been several attempts at this topic, yet they are still singular and awaiting improvements, such as increasing throughput and accuracy. The most promising are label-free approaches, such as tracking the nanometer membrane fluctuations of live cells during molecular interactions, which can provide insights into the binding kinetics and strength of different molecules.^67^ Another method is second harmonic light scattering (SHS), which can be utilized to quantitatively monitor the adsorption and transport of molecules across membranes in living cells, providing real-time information on molecular interactions at the plasma membranes.^68^ In-cell NMR-based approaches have been developed to monitor DNA-ligand interactions inside the nuclei of living cells, allowing for the assessment of ligand behavior in the intracellular environment.^69^ Avoiding labeling allows for the observation of processes without external influence. However, these can be limited to only a few processes with sufficient signal-to-noise ratios.

An alternative is the labeling of substrates or products by detectable tags. The most popular are fluorescent tags, though Raman tags are also gaining interest.^70^ For fluorescent tags, FRET seems to be the most convenient technique for studying reactions. Several studies have utilized FRET in living cells for various applications. For example, a gene-encoded FRET sensor was developed to monitor the action of ATG4B during autophagy in living cell.^71^ Sun et al. developed a FRET sensor to detect arginine methylation levels *in situ*.^72^ Another work presented the study on protein–protein association dynamics based on FRET.^73^ Phillip et al. combined genetically encoded labeled protein with a microinjected bioconjugate. By principle, the FRET technique involves using two fluorescent dyes, each needing a separate experimental procedure to be introduced to living cells. There are strategies for reducing the number of labels. For example, FCS of single labeled molecules in cells can be used to determine the kinetic parameters of biochemical reactions. Kwapiszewska et al. studied diffusion coefficients of Drp1 protein homo-oligomers, providing *K*_D_*f*or tetramerization.^59^ In another work, Karpińska et al. used FCS for monitoring Olaparib drug association with its targets in breast and cervix cancer cells.^61^

## Technological Challenges

Quantifying cellular processes poses a considerable challenge, primarily due to the intricate nature of the cellular interior. The variability in molecule quantities within a seemingly homogeneous cell population further complicates this task for various cellular processes.^14^ This variability undermines the extrapolation of data acquired at the single-cell level to entire cell populations. One potential approach to address this challenge is to augment the sample size, which often comes at the cost of extended time or diminished sensitivity.

The central challenge in developing methods for quantifying biochemical reactions lies in balancing throughput and sensitivity. Prioritizing sensitivity is crucial when dealing with a few molecules in a highly complex matrix. Subsequently, efforts should focus on increasing throughput at various stages, encompassing hardware automation for data acquisition and software development for processing and analysis. This delicate equilibrium is essential for achieving reliable and meaningful quantitative values for biochemical reactions.

Bioanalytical methods are rapidly evolving to tackle the current challenges in accurately mapping biochemical interactions in living cells. The focus is on developing innovative technologies that enhance the sensitivity and reliability of measurements. Fluorescence-based methods are particularly promising for single-molecule studies *in vivo*, but the challenge lies in overcoming background autofluorescence within the cellular interior.^62^ To achieve this, labeling and detection methods must be developed to enhance signal-to-noise ratios and improve tagging strategies. Moreover, the multichannel mode of fluorescent imaging enables the simultaneous observation of multiple parameters. These improvements will be crucial for unlocking the full potential of label-based bioanalytical techniques in the future. In parallel, there is a significant need for label-free methods to distinguish the analyte of interest from the complex intracellular matrix. Avoiding labels is crucial for observing native intracellular processes^67,74^ without any factors that potentially can influence their progress.^75^

Additionally, future bioanalytical methods should aim to bridge the gap between spatial and temporal resolutions, pushing the limits of spatiotemporal resolution for direct observation of biochemical reactions *in vivo*. Modern light microscopy can provide a spatial resolution of below 10 nm^43^ or a temporal resolution of 88 million pixels per second.^76^ However, these two limits cannot be achieved simultaneously. The awaited breakthrough lies in technologies that seamlessly couple spatial and temporal resolution, offering unprecedented insights into living cells’ dynamic and intricate processes.

Improvement in experiments’ biochemical and physical aspects should be accompanied by a focus on data processing and the interplay between instrumentation and new software tools. In the future, data analysis software will need to work in a multidimensional space since the parameters of biochemical reactions depend on various intracellular environment parameters, molecular pathway network reactions, and cell-to-cell variability. Handling all these quantities simultaneously will require powerful tools. Computational techniques like quantum computing, distributed computing, and edge computing can benefit biochemistry *in vivo* significantly.^77^ Graph database technology is another promising tool that can help handle relationships between data points in multidimensional spaces. Advanced artificial intelligence (AI) and machine learning (ML) are inseparable elements of data processing development.^78^ As AI and ML become more critical, there is an increasing emphasis on developing explainable and interpretable models, called Explainable AI (XAI).^79^ Advancements in storage technologies, such as nonvolatile memory (NVM) and persistent memory, contribute to faster data access and retrieval times. This is essential for handling large data sets efficiently.^80^

In parallel, developing theories and simulations is also an essential part of research on biochemical networks and biological heterogeneity. *In silico* modeling is capable of genome-scale simulations and predictions of metabolic changes in microorganisms.^81,82^ The differentiation of cells in multicellular organisms could also be explained via modeling.^83^ Above all, supporting interdisciplinary approaches will be necessary. Collaborations between experts in various fields, including computer science, mathematics, and domain-specific areas, lead to innovative solutions for processing and interpreting multidimensional data.

## Future Questions

The interior of living cells is incredibly complex and mainly off-limits to biophysical and biochemical research. However, the development of bioanalytical technologies is expected to broaden the possibilities of collecting reliable, quantitative data on cellular processes in their natural environment. This will be an exciting new area of research that will allow scientists to compare existing *ex vivo* data with *in vivo* data. This new research area will raise many questions, such as the reliability of measurement methods and the influence of different factors on bioprocesses. For instance, biochemists are interested in the impact of macromolecular crowding on reactions that occur *in vivo*. However, researching this topic *ex vivo* has proven to be highly challenging. One strategy is to use artificial crowders like polymers. However, their contribution to the studied reaction can go beyond steric restrictions, as adding polymers to the buffer can significantly change ionic strength and affect biomolecule conformation.^8^ Thus, isolating the influence of only one factor seems to be an exceptionally difficult task.

Tracing intracellular reactions is a complicated task due to the complexity and diversity of molecular networks. Even with the unprecedented development of molecular biology, many factors are still waiting to be discovered, and we continue to learn about the connections and interactions between biomolecules. From a technical standpoint, tracking one reaction at a time seems practical. Still, given the heterogeneity of cellular populations, it cannot be assumed that only one process is affected in a given study. As a result, there is a vast demand for multiplexing and increasing throughput, both in hardware and software, to extract reliable data. Hopefully, these advanced strategies will result in the discovery of new, unknown intracellular interplays.

The question is to what extent biological heterogeneity depends on stochastic or environment-affected processes. Every cellular process occurs at its core when biomolecules come close, match their structures, and react. This can be influenced by physical and chemical factors such as ion composition, temperature, and viscosity, which were broadly discussed above. This raises the question of whether fluctuations in these quantities can be externally regulated or are mainly regulated by molecular proportions. A better understanding of the interplay between physical factors and their biological outcomes will provide more insight into this topic. We await solutions that will allow for simultaneous intracellular monitoring of many factors, both environmental and those related to the reaction of interest. With this, we can identify which factors affect the process and determine whether they can be regulated externally or intrinsically. This will improve experimental design and allow for the division of populations of organelles/cells/organisms with a broad spectrum of heterogeneities into well-defined, narrower subpopulations.

## Conclusions

Biological populations exhibit a great deal of diversity, which results in several advantages, including biodiversity and gene pool enrichment within species. However, it also brings about some drawbacks. For instance, some individuals may not respond to medical treatments or may experience severe adverse effects. As a result, identifying and comprehending factors that lead to such diverse responses to external factors can significantly enhance the field of medicine.

All biological processes originate from the reactions of biomolecules, which obey the laws of thermodynamics and chemical kinetics. There are numerous experimental techniques to observe and quantify these reactions in living cells. However, obtaining multiparameter data with adequate spatiotemporal resolution still requires a technological breakthrough. Developing new measurement and data processing techniques will lead to further research fields, and combining physical chemistry with molecular biology will provide novel insights for both research areas.
